# Surgical strategy of urachal remnants in children

**DOI:** 10.1093/jscr/rjz222

**Published:** 2019-07-23

**Authors:** Yukihiro Tatekawa

**Affiliations:** Department of Pediatric Surgery, Saku Central Hospital Advanced Care Center, 3400-28, Nakagomi, Saku-shi, Nagano, Japan

## Abstract

A urachus is a vestigial tubular structure that connects the urinary bladder to the allantois during early embryonic development. Urachal remnants are classified as patent urachus, urachal sinus, urachal cyst, and urachal diverticulum. Ten patients with urachal remnants underwent surgery at our institution between 2015 and 2019. Six patients had a urachal sinus, and four had a urachal diverticulum. Two patients with urachal sinus underwent excision of the urachal remnant, from the umbilicus to the urinary bladder, using an umbilical approach. The other four patients with urachal sinus underwent laparoscopic surgery with excision of the urachal remnant, from the umbilicus to the urinary bladder. All patients with urachal diverticulum underwent open excision of the diverticulum through a Pfannenstiel incision. Pathologic examination of all urachal remnants showed no evidence of neoplasm and complete excision. All patients had an uneventful postoperative course and are doing well.

## INTRODUCTION

The urachus is a vestigial tubular structure that, in early embryonic development, connects the urinary bladder to the allantois. These remnants may be divided as either patent urachus (communication between the umbilicus and bladder), urachal sinus (umbilical end is open but there is no communication with the bladder), urachal diverticulum (forms a cap on the dome of the bladder), and urachal cyst (central part of the tract is patent and fills with fluid) [1]. Carcinoma of the urachus has been reported [2]. There are many recent reports of laparoscopic excision of urachal remnants [3–5]. In this case series, we evaluate the surgical strategies for addressing urachal remnants in children.

## CASE SERIES

Ten patients underwent surgery for a urachal remnant at our hospital between 2015 and 2019. A total of six patients had a urachal sinus, and four had a urachal diverticulum. Two of the patients with a urachal sinus underwent excision of the remnant, from the umbilicus to the urinary bladder, using an umbilical approach. The other four patients underwent laparoscopic surgery with excision of the urachal sinus, from the umbilicus to the urinary bladder. All four patients with a urachal diverticulum underwent excision through a transverse suprapubic (Pfannenstiel) incision.

### Surgical method 1: umbilical approach to a urachal sinus

**Figure 1:**
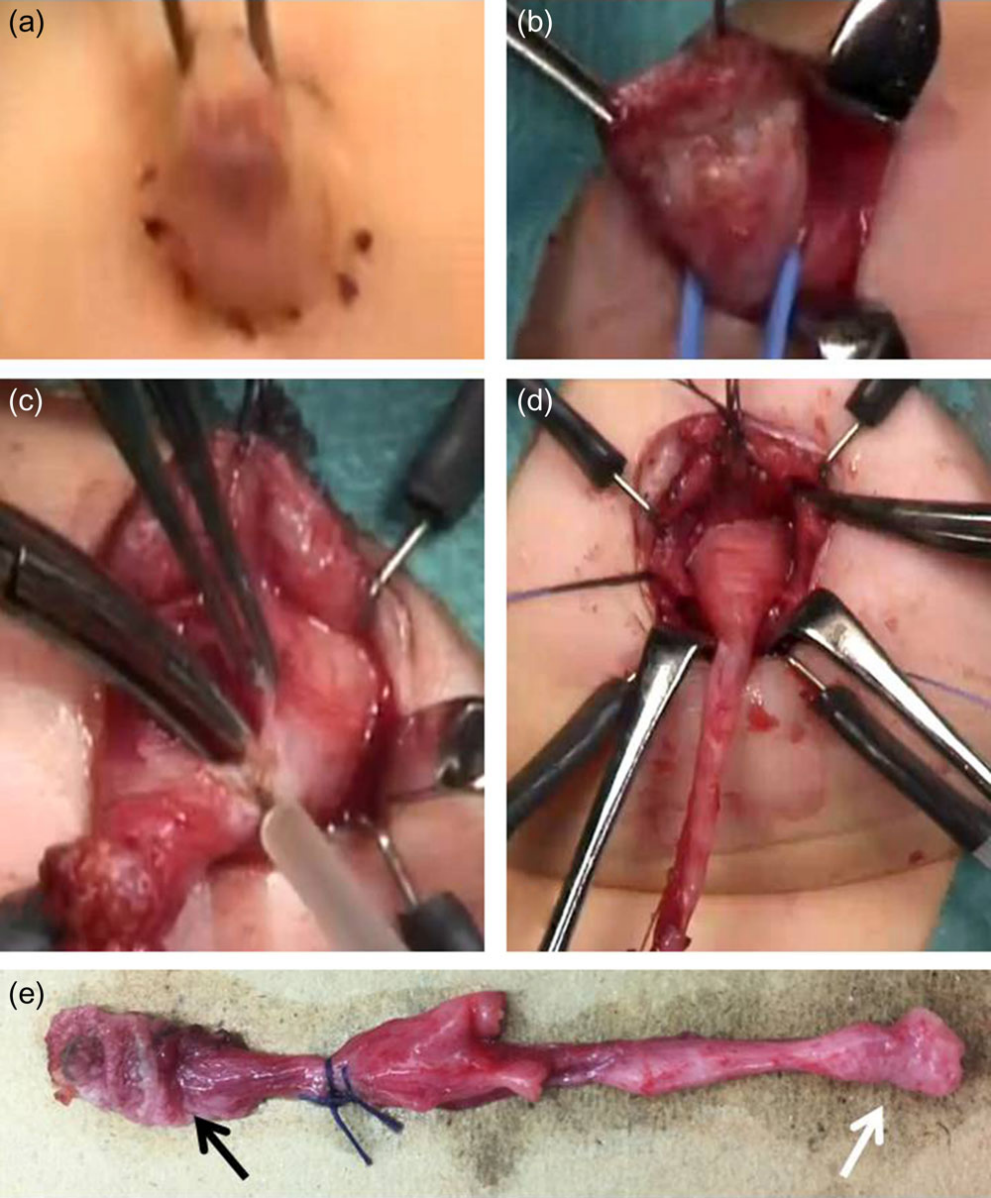
Surgical method 1. (a) Incision under the umbilical ring. (b) Entire resection of the fistula. (c) The umbilical edge of the urachal remnant is separated and the linea alba is divided in a T-shape. (d) The bladder cuff, including the urachal insertion site, is excised en bloc with the entire urachal sinus. (e) The resected specimen reveals a urachal remnant (black arrow: urachal sinus; white arrow: bladder wall).

The umbilicus was incised under the umbilical ring (Fig. 1a), and the fistula was wholly resected (Fig. 1b). The umbilical edge of the urachal remnant was separated from the umbilical base, and the linea alba was divided from the level of the umbilicus in a caudal direction using a T-shaped incision (Fig. 1c). The bladder was filled with saline through a urethral catheter, and a bladder cuff was excised, including the urachal insertion site en bloc with the entire urachal sinus (Fig. 1d and e). In both patients that underwent this procedure, pathological examination of the resected specimen showed a urachal remnant in the umbilical mass or at the stump on the bladder side.

### Surgical method 2: laparoscopic excision of a urachal sinus

**Figure 2:**
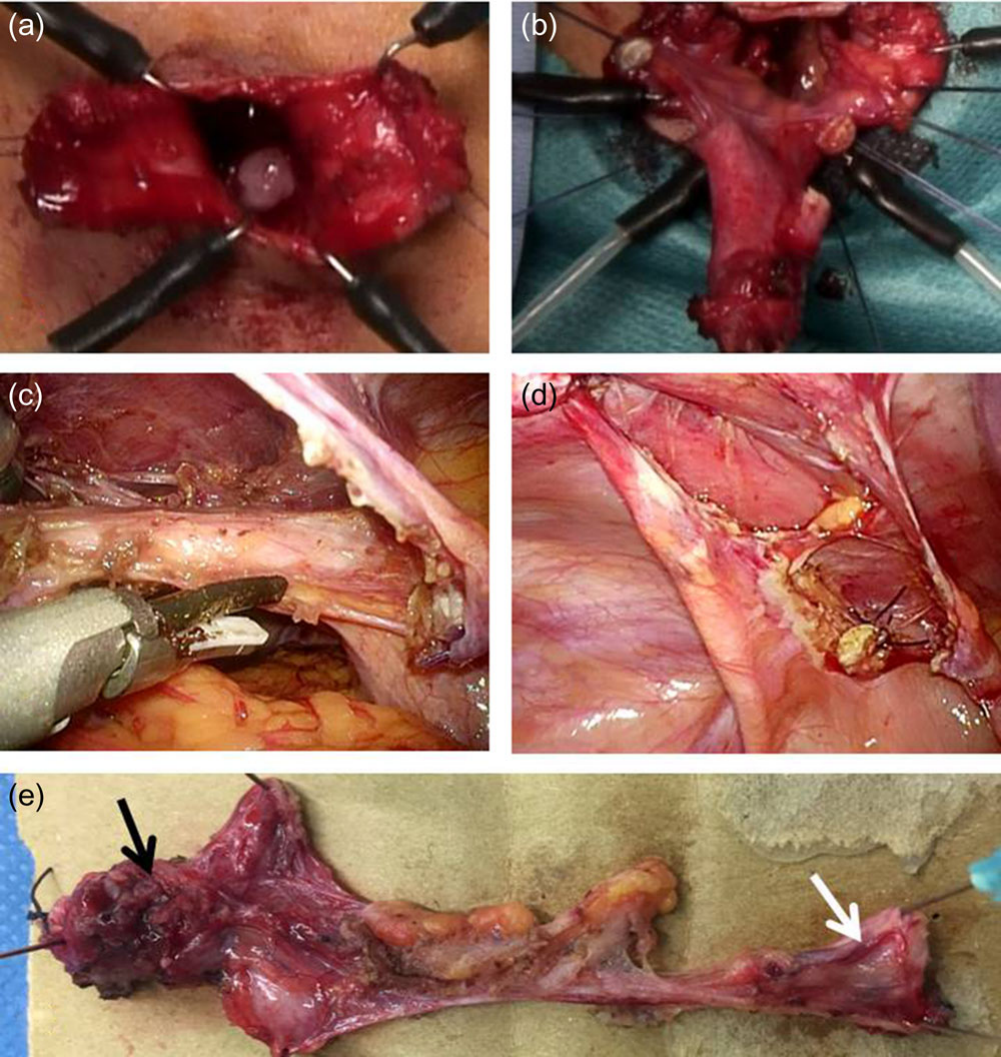
Surgical method 2. (a) The umbilicus is vertically divided to create a pair of lateral skin flaps, and the fistula is entirely resected. (b) The umbilical edge of the urachal remnant is separated, and the linea alba is divided in a T-shape. The partially resected urachal sinus is dropped into the abdominal cavity. (c) and (d): The bladder cuff, including the urachal insertion site, is ligated using an endoloop at the dome. The urachal remnant is resected at the dome of the bladder. e: The resected specimen reveals a urachal remnant (black arrow: urachal sinus; white arrow: bladder wall).

After establishing pneumoperitoneum, the umbilicus was divided vertically to create a pair of laterally based skin flaps, and the fistula was wholly resected (Fig. 2a and b). The linea alba was divided from the level of the umbilicus in a caudal direction, and the partially resected urachal sinus was dropped into the abdominal cavity. An Alexis XS wound retractor (Applied Medical Resources Corporation, Rancho Santa Margarita, CA) was inserted through a vertical transumbilical incision, and a silicone cap (Free Access; TOP Corporation, Tokyo, Japan) was placed for use as a multichannel port. A 5-mm port for the flexible laparoscope was inserted through this cap. Two additional 5-mm ports were inserted to enable use of a grasping forceps and an ultrasonically activated scalpel (Fig. 2c). The bladder was filled with saline through a urethral catheter, and a bladder cuff, including the urachal insertion site, was ligated at the dome using an endoloop (Ethicon PDS II Endoloop® Ligature or Ethicon Vicryl Endoloop® Ligature; Ethicon US, LLC.) (Fig. 2d). The urachal remnant was resected at the dome of the bladder (Fig. 2e). In three of the four patients that underwent this procedure, pathologic examination demonstrated a urachal remnant; only connective tissue was identified in the fourth patient.

### Surgical method 3: excision of a urachal diverticulum through a Pfannenstiel incision

**Figure 3:**
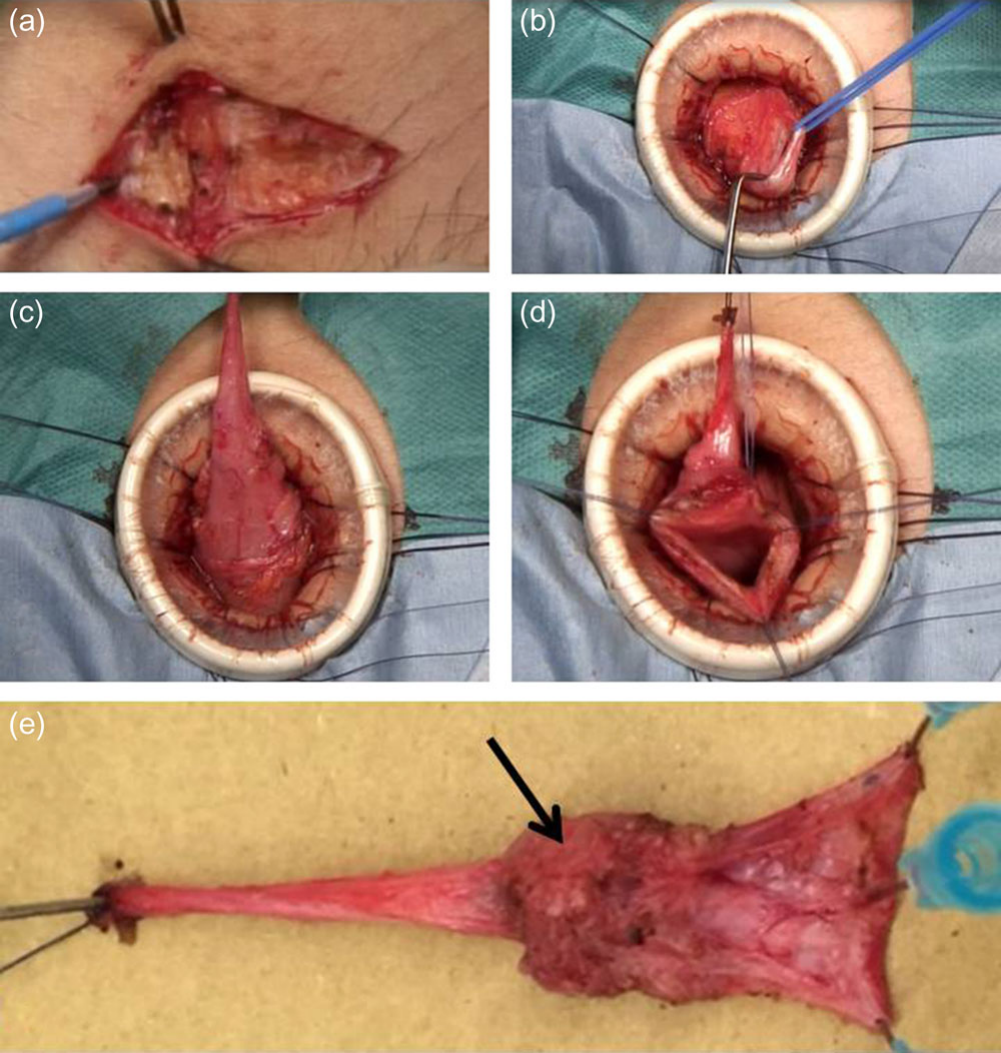
Surgical method 3. (a) A Pfannenstiel skin incision and a vertical midline fascial incision are made. (b) The cranial side of the urachal remnant is exposed and resected. (c and d): The urachal diverticulum is exposed and resected at the dome of the bladder, including resection of the bladder wall. (e): The resected specimen reveals a urachal remnant (black arrow: urachal diverticulum).

Using a transverse suprapubic Pfannenstiel skin incision and a vertical midline fascial incision, the surface of the bladder was exposed and an Alexis XS wound retractor was inserted (Fig. 3a). The bladder was filled with saline through a urethral catheter, and the urachal remnant was continuously dissected as far as possible proximally toward the umbilicus, and then resected (Fig. 3b and c). The urachal diverticulum and a portion of the bladder wall were resected at the dome of the bladder (Fig. 3d and e). The opening at the bladder apex was closed under direct visualization using absorbable suture. In all four patients, pathologic examination confirmed the presence of a urachal remnant.

In surgical methods 1, 2 and 3, all the patients had a urethral catheter, left in after surgery for 2 days.

### Follow-up

All 10 patients underwent an uneventful postoperative course and were discharged from the hospital in a timely fashion.

## DISCUSSION

When the distance between the umbilicus and the dome of the urinary bladder is short, as it is in infants, surgical method 1 may be suitable for resecting a urachal remnant. However, it was necessary to expand the urinary bladder by injecting saline through a urethral catheter. Surgical method 2 was performed mostly in between 7 years old and 15 years old. Our experience is consistent with the recommendations of Naiditch *et al.*, who state that the umbilical approach is appropriate for infants and laparoscopic surgery is recommended for older children [6].

We actively excise urachal remnants to prevent recurrent infection and the long-term risk for malignancy. However, there are reports that describe conservative therapy [1, 6, 7]. Some patients with urachal remnants experience spontaneous resolution. In particular, patients younger than 1 year of age seem to experience resolution with nonoperative management. However, we saw a 2-month-old boy who developed an infection and underwent resection of a urachal remnant using surgical Method 1. It may be necessary to perform surgical resection to prevent recurrent infection, even if the patient is younger than 1 year of age.

Many reports concerning the management of urachal remnants are available, describing either surgical resection or conservative therapy. It is necessary to follow patients every 3–6months for five years when there is the possibility of incomplete excision or carcinoma of the urachus [8, 9].
